# Investigating the potential of oncolytic viruses in the treatment of melanoma: where do we go from here?

**DOI:** 10.1093/skinhd/vzaf022

**Published:** 2025-04-22

**Authors:** Michaela Houghton, Annwyne Houldsworth

**Affiliations:** Faculty of Health and Life Sciences, University of Exeter, Exeter, UK; Faculty of Health and Life Sciences, University of Exeter, Exeter, UK

## Abstract

Oncolytic viruses (OVs) can destroy cancer cells without harming healthy cells. This review explores the mechanisms by which OVs operate and the methods of delivering them. Melanoma is a common type of skin cancer with increasing prevalence in the UK; therefore, finding effective strategies to combat the disease is paramount. To understand the potential of OVs in treating melanoma, different types of viruses will be reviewed. Talimogene laherparepvec (T-VEC) is the only OV to be approved for treating melanoma; this review aims to understand the efficacy of T-VEC as a monotherapy and combined with other treatments. There is substantial evidence to support the use of OVs in treating melanoma by synthesizing the current perspectives of their use where they proved to be effective in clinical trials, as monotherapies and in combination with other treatments, as well as exciting innovative ventures using novel virus species. Gaps are also highlighted in the research, such as determining the influence that cancer gene mutational status has on how the tumour cells react to treatment, a concept that should also be considered in future research.

## Teaser

Melanoma is a common type of skin cancer that may be successfully treated with oncolytic viruses. Innovative gene transformation of novel viruses are able to infect cancer cells and allow the host to mount an inflammatory immune response to the tumour. Combination therapies, using factors that stimulate the cytotoxic response to cancer and gene therapy, can be combined with this kind of immunotherapy. Where will it take us next?

Melanoma is the fifth most common type of cancer in the UK, with 17 500 new cases each year between 2017 and 2019, and more than 25% of cases being diagnosed in people under the age of 50 years, and is becoming increasingly common.^1^ In 2022, 60 000 people died of the disease worldwide.^2^ QIMR Berghofer Medical Research Institute estimated that a cost of $201 million would be needed to treat melanoma in 2017, demonstrating that this is a global issue with a huge economic burden globally, which remains a major public health threat.^3^

Increased exposure of vulnerable light skin in White populations to ultraviolet (UV) light presents a strong risk factor for melanoma and there has been a marked increase in the incidence of this disease in recent years within populations of European heritage.^4^ The thinning of the earth’s ozone layer has increased human exposure to higher levels of UV radiation and may be a causal factor for the significant increase in skin cancer recently.^5,6^

If left untreated, melanoma can metastasize to other sites across the body, and with advancing disease progression, the 5-year survival rate after diagnosis declines from 70% at stage III to 30% at stage IV.^7^ Oncolytic virotherapy describes the therapeutic use of viruses to infect, selectively replicate within and kill cancer cells, without impairing healthy cells. Oncolytic viruses (OVs) can be genetically engineered or utilized in their natural form. In 1904, the description of a patient with leukaemia who entered temporary remission after experiencing a presumed influenza infection in 1896 laid down the foundation for the conceptualization of utilizing OVs as cancer therapy.^8^ Over 100 years later, in 2015, the first OV was approved for therapeutic use when the United States Food and Drug Administration (FDA) launched its recommendation for the use of talimogene laherparepvec (T-VEC), a herpes simplex virus, in treating patients with melanoma with accessible tumours that are unresectable.^9^ The FDA approval of T-VEC use as a monotherapy for melanoma does not define the limits of the potential of OVs, rather, it sets the precedent for their development and improvement.^10,11^

This review explores some areas of research and treatment development of current and proposed therapies for melanomas in addition to combination strategies, such as, T-VEC OV therapy and immune checkpoint inhibitors ICIs.

## Melanoma pathogenesis

Melanoma is a type of skin cancer that develops from skin cells called melanocytes, pigment-producing cells at the basal layer of the epidermis. Specialized organelles called melanosomes produce the pigments pheomelanin and eumelanin and prevent UV-induced DNA damage.^12^

There are different types of melanomas, including superficial spreading, nodular, lentigo, amelanotic and aural, which develop differently and at different rates, each with a separate pathophysiology, colour and form.^13^

Melanocytes develop from neural crest-derived cells with some shared transcription factors that contribute to melanoma plasticity.^7,14,15^ There are molecular changes to cells resulting in heterogeneous accumulation of adherent cells. Melanocytes originate from the embryonic ectoderm germ layer, but the tissue-invasive phenotype of melanoma cells undergoes dedifferentiation, changing through different transcription programmes, to acquire a new genetic plasticity, in a similar way that epithelial cells dedifferentiate and transition into mesenchymal cells. The phenotype switching and plasticity of tumour cells enable them to proliferate and evade the host immune system. These adaptions and changes to the tumour microenvironment (TME) enable disease progression and drug resistance.^16^ This is an important factor in the immune escape and metastasis of melanoma cancer cells.^17,18^

Genetic and epigenetic changes in cancer cells can cause them to multiply and grow abnormally. Tumour suppressor genes are oncogenes that can cause a loss or gain of function. Some mutations are due to chemicals, ageing or UV light. Hypomethylation of DNA patterns can occur as epigenetic changes as well as histone modifications that may change gene expression.^19^ Another change that can occur is in chromatin structure where the regulatory structures of the genome are affected.^20^ The genetic mutational status of melanoma^21^ can influence the applicability of treatment options. The human oncogene *BRAF* is involved in the control of cell growth, encoding the B-raf protein. Some cancers have the V600E mutation, which results in uncontrolled cell division and growth of cells. This mutation confers a poor prognosis for cancer patients.^22^ Another gene that promotes cell growth, maturation and survival is *MAP2K1* (mitogen activated protein kinase kinase). *MAP2K1* plays a role in the development of human cancers and is a transducer of a growth factor receptor.^23^

There are different types of melanomas that have been identified, including superficial spreading, nodular, lentigo and aural, which develop differently and at different rates, each with a separate pathophysiology, colour and form.^13^

## Tumour immune microenvironment

The tumour TME has a direct effect on the immune response of cells and involves the expression and activity of cytokines, such as interleukins, interferons and growth factors, like transforming growth factor (TGF)-β, vascular endothelial growth factor and epidermal growth factor (EGF). The modulation effects of these signalling pathways when combined with OVs may influence and inhibit the progression of cancer cells. Genetic engineering of these inhibitory factors either as cis or trans factors in the design of the OV or in combination with monoclonal or bispecific antibodies provides encouraging opportunities for novel approaches in cancer treatment development.^24,25^

## Current treatments for melanoma

Surgical resection of the primary tumour serves as the first line of defence employed in treating melanoma; if surgery alone is not viable, therapeutic strategies such as chemotherapy, radiation therapy, targeted therapy and immunotherapy may be required.^26^

The use of neoadjuvants as pretreatment therapies to surgery to shrink the tumour, using radiation or hormone therapy or immunotherapy, has been shown to achieve high response rates and recurrence-free survival rates in stage III melanoma.^27^

One first-line treatment for BRAF mutation-positive melanoma is the use of a BRAF kinase inhibitor called vemurafenib.^28,29^ Despite the clinical efficacy and rapid action of vemurafenib, it is limited in its short duration of response and significant skin toxicities;^28^ these factors highlight an unmet clinical need for an effective therapeutic method to combat advanced melanoma. There are three approved melanoma combined BRAF/MEK inhibitors (BRAFi/MEKi): encorafenib with binimetinib, dabrafenib with trametinib and vemurafenib with cobimetinib. Encorafenib in combination with binimetinib is used to treat melanoma that has spread or cannot be surgically removed. However, some toxicities are reported and monotherapies have different side-effects to combination therapies.^30^

Unfortunately, some patients experienced multifocal choroiditis after treatment with BRAFi/MEKi, indicating some ocular toxicity and a risk of visual impairment.^31^

The current issues pertaining to advanced melanoma treatment include targeting brain metastases, overcoming development of resistance to targeted and immune-based therapies and diminishing toxicities.^32^ Whether or not OVs can fulfil this demand is yet to be comprehensively determined.

The immunotherapeutic use of OVs could pave the way to revolutionizing cancer treatment, especially in advanced melanoma. Therefore, it is imperative to understand the mechanisms by which OVs operate, the impact that delivery methods have on their efficacy, the types of viruses that can be used to target melanoma, and the contrast between monotherapy and the use of OVs in combination with existing treatments, all of which will be addressed in this review. The aim of this review is to determine the potential of OVs in treating advanced melanoma.

## Immune checkpoint inhibitors

One important area that has been transformative in the development of cancer therapy is in the understanding of the tumour TME with differences in apoptotic signals, such as programmed cell death receptors and ligands (PD-1/PD-L1). The immunosuppressive ICI pathways and other immune inhibitory factors, like cytotoxic T lymphocyte associated protein (CTLA4), are significant targets to change the TME. There are also changes in the expression of major histocompatibility factor and T-cell receptors in cancer cells, which enables the tumour to hide from the immune system. A recently identified factor in tumour cells is the enzyme CD73, which is highly expressed in some tumour cells that are resistant to the host immune system. Some promise has been observed in immunotherapy preclinical trials that inhibit immune checkpoint factors, making the tumour more visible to inflammatory responses. Some positive outcomes of ICI inclusion of systemic treatment for melanoma have been well tolerated with good patient satisfaction.^33^

The combination of OVs with ICI for advanced melanoma is well tolerated in patients with melanoma but the perfect engineering of inhibitory genomics is still being developed.^34^

Monoclonal antibodies directed at these ICIs are able to reverse the TME changes caused by the tumour and several have been approved for the treatment of several tumours.

Ipilimumab and nivolumab are CTLA4 and PD-1 inhibitors, respectively, and can enhance an inflammatory response by reversing the cancer immunosuppressant environment, also enhancing the natural killer (NK) cell activation against the tumour.^35,36^

## Viral tropism

Several factors affect virus attachment, entry, replication, viral assembly and budding. There is also some heterogeneity in viral replication cycles and host heterogeneity in viral entry receptors required for viral attachment.^37^ Viruses can have non-genetic-encoded pleomorphic virion shapes and structures that can influence the host cell attachment and immune response to the virus.^38^ Exploiting this concept of virus pleomorphism could be an advantage in genetically engineering the design of OVs, enhancing the survival of the OV and enabling the virus-infection of the tumour cells to persist and continue to be recognized by the host immune system until the tumour has sufficiently diminished.

For a virus to infect a specific host species, tissue, organ or cell type, it has to cross different types of barriers to infect its target. The ability of the virus to attach to cellular receptors is a key factor to the success of infection.^39^ An example is where HIV infects macrophages not neurons. The success of viral replication can be affected by the host pH or temperature and in addition to these obstacles, the host immune response has a significant impact on the success of viral infection.^40^ In particular, the presence of host proinflammatory cytokines can have an influence on the success of infection, including some polymorphic genetic variants in some individuals that can determine the successful infection by the virus.^39,41^

It is important that the intracellular environment is able to enable viral replication, depending on cell transcription factors, organelles and the activation state of the host cell.^42^ There are several other factors that can affect host tropism, including species-specific infections; however, many examples of zoonotic transfer of viruses jumping from one species to another has caused some serious concerns in recent years. Some viruses target specific organs or tissues and can be engineered to target specific tumours,^40^ an example being that influenza virus infects lung tissue but not brain tissue.

The site of entry of a virus can determine its successful infection of a host and host-cell receptors can be a specific target for viruses to enter host cells where some viruses exhibit cellular tropism by only infecting one type of cell.^43^ Targeted infection of epithelial cells lining lymphatic and blood vessels, where the vasculature and architecture of tumour tissue is disrupted, causing blood clots by an OV infection can result in the tumour losing its neovascular blood supply.^44^ Viral targeting of glycocalyx of the target tissues can result in vascular permeability.^45^

## Tumour cell death

There are several mechanisms of OV-induced cell death,^46^ and a spectrum of different cell death modalities exist between the metabolically efficient apoptosis and cellular disintegration of necrosis, including as necroptosis, pyroptosis and ferroptosis.^47,48^ Different types of immune responses are induced with different distinct versions of cell death.^49^ The distinct programmed cell death known as apoptosis is an intrinsic event involving mitochondrial pathways, whereas other extrinsic pathways can occur through death receptor pathways.^50^ Apoptosis is often associated with tolerogenic cell death but depending on the damage-associated molecular patterns (DAMPs) released, it can be tolerogenic or immunogenic.

Phagocytosis of the end products of cell death can evoke immunogenicity or tolerogenicity.^48,51^ Non-apoptotic mechanisms are immunogenic and associated with uncontrolled inflammation.^51^ Necroptosis is a regulated from of necrosis, morphologically similar to necrosis and triggers an inflammatory response in surrounding tissue to the tumour. Similarly pyroptosis is a programmed form of necrosis where the cell contents are released in surrounding tissue and elicits an inflammatory response.^52,53^ Ferroptosis is dependent on iron and lipotoxic, where a build-up of reactive oxygen species is characteristic of this form of cell death.^54^

OVs can induce an immunogenic tumour cell death and sometimes promote a long-lasting antitumoral immunity.^48^ Pathogen-associated molecular patterns (PAMPs) and DAMPs, as neoantigens, can be exposed in this process, and they promote these inflammatory reactions.^51^

### Mechanisms by which oncolytic viruses act on tumour cells

OVs are able to infect tumour cells through nuclear transcription factors that are selectively expressed by the tumour cells, acting as receptors for the virus or enabling viral gene expression.^55^ There are three mechanisms by which OVs operate to kill tumour cells: direct oncolysis or inducing apoptosis of the infected tumour cell,^56,57^ stimulating apoptotic death of surrounding uninfected cells^57^ and initiating an immune response.^58^ Immunostimulants of the TME can enhance the immune response against tumour cells. For example, proinflammatory stimulatory cytokines like interleukin (IL)-12 can be inserted into the genome of the oncolytic virus, and it was found that introducing a fusion peptide was superior to coexpressing subunits of IL-12.^59^

**Figure 1:**
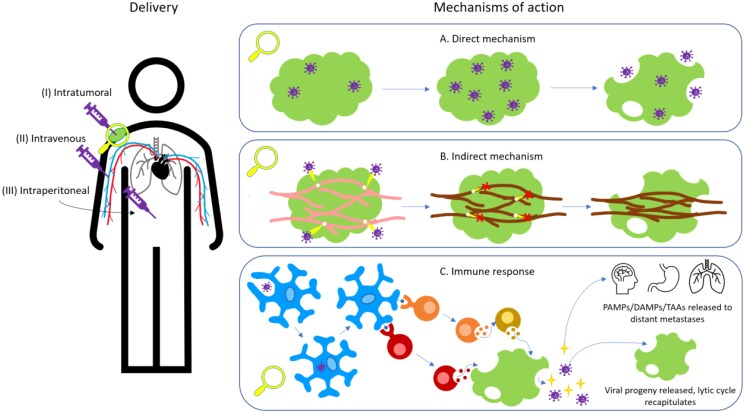
The main administration routes used to deliver oncolytic viruses (OVs) in patients: (i) intratumoral delivery whereby the OV is injected directly into the tumour; (ii) intravenous delivery, which entails administration of the OV via the veins; (iii) intraperitoneal delivery, which involves administering the OV into the abdominal cavity. Zooming in, the mechanisms by which OVs operate in destroying tumour cells is presented. (a) In the direct mechanism, OVs recognize tumour cells through signals expressed in the tumour microenvironment (TME), entering the tumour cell through various receptors, instigating an infection of the host cell. Thereafter, the OV begins replicating utilizing its cellular machinery, generating viral proteins, impairing host cell function, inducing oxidative stress and sparking the activation of apoptotic pathways. (b) From the indirect approach, certain OVs have genes encoding neutrophil chemoattractants, which results in the recruitment of neutrophils, which diminish the vascular perfusion of the tumour vasculature, thus indirectly triggering hypoxia and apoptosis in the tumour cells. (c) In initiating an immune response to the tumour cells, antigen-presenting cells endocytose some viruses, where smaller viral particles are processed within the cell. The antigen-presenting cells express the viral proteins activating the stimulation of natural killer (NK) cells, CD4^+^ T cells and CD8^+^ T cells which launch a direct attack on tumour cells. This results in the destruction of tumour cells, releasing viral progeny along with pathogen-associated molecular patterns (PAMPs), damage- associated molecular patterns (DAMPs) and tumour-associated antigens (TAAs), allowing the cycle to continue in other tumour cells. Adapted from the information provided in Kroemer *et al*.^48^ and Jiang and Fueyo.^62^

Direct oncolysis refers to the ability of a virus to lyse or induce apoptosis in the host tumour cell, as described in Figure 1.^60,61^ In initiating an immune response to the tumour cells, antigen-presenting cells endocytose some viruses, where smaller viral particles are processed within the cell. The antigen-presenting cells express the viral proteins activating the stimulation of NK cells, CD4^+^ T cells and CD8^+^ T cells, which launch a direct attack on tumour cells. This results in the destruction of tumour cells, releasing viral progeny along with PAMPs, DAMPs and tumour-associated antigens, allowing the cycle to continue in other tumour cells.^48,62^

The virus replicates within the tumour cell, destroying it, and then infects neighbouring tumour cells where the lytic cycle is recapitulated until it is attenuated by an immune response, or the number of susceptible host cells has depleted.^61^ Defects in the apoptotic pathway, such as p53, are present in many tumour cells, propagating tumour cell proliferation.^48,63^ OVs can be altered to circumvent this through deletion of the viral protein E1, which is an inhibitor of apoptosis, thus increasing p53 and triggering apoptosis.^63,64^

The indirect mechanism operates by triggering apoptosis in neighbouring uninfected cells of the TME.^57^ OVs can be designed to target the vasculature supplying the tumour cells thus impairing tumour angiogenesis and reducing blood flow to the tumour cells and causing tumour hypoxia.^65,66^ As a result, the tumour cells become infected and necrosis occurs, as shown in Figure 1.^65^

Finally, the immunosuppressed TME can become highly immunogenic, with viral infection spurring the production of cytokines, chemokines and the release of tumour antigens from the lysed tumour cells, thus initiating a systemic immune response to uninfected tumour cells.^67^ Figure 1 shows how a systemic immune response can then be propagated to target distant and uninfected lesions elsewhere in the body.^68^

### Delivery methods for oncolytic viruses

As visualized in Figure 1, OVs can be administered intratumorally, with the advantage of maintaining the concentration of virus administered, diminishing the likelihood of misdirection of the virus to other organs thus achieving greater therapeutic efficacy.^69–71^ Researchers have found that intratumoral delivery allows for more precise control of the concentration of virus being administered, enhancing the generalizability of *in vitro* experiments to *in vivo* application.^72–74^ Intratumoral delivery meets limitations in reaching deeper lesions or inaccessible tumours,^75–78^ like prostate cancers, and repeat dosing for less accessible tumours presents a challenge owing to the complex procedures.^79,80^ Melanomas are usually more accessible for this type pf delivery. However, not all primary melanomas are visible on the skin, as 4–5% are noncutaneous – they can be located in ocular (eye), mucosal (of the mucous membranes), oral (mouth), anal or rectal, vulvar, vaginal, nasal sinuses or other mucosal linings.^81^

Alternatively, OVs can be delivered systemically by means of intravenous or intraperitoneal administration, which is displayed in Figure 1, overcoming the obstacle of reaching inaccessible tumours, including bypassing the blood–brain barrier to target brain tumours.^76^ Intraperitoneal administration of OVs is found to absorb faster than subcutaneous injection and is easier to perform, with the added benefit of being an ideal route for targeting tumours located within the abdominal cavity,^82,83^ falling short in that it is absorbed slower than in intravenous delivery.^82^ Intravenous delivery faces hurdles in its vulnerability to being eliminated by the immune system as well as being the most likely route to result in toxicity.^82^ In addition, different modes of delivery are important to consider, as most melanoma tumours can be injected topically with the viral immunotherapy but combinations of systemic delivery by infusion and injections can penetrate deeper into solid tumours.^84–86^ Currently, intratumoral injection has been a convenient mode of delivery but is diluted by this method affecting bioavailability.^87^

Common side-effects from intratumoral injection of OVs for melanoma include injection-site pain with inflammation, and flu-like symptoms, such as, chills, fatigue, muscle aches, pyrexia and nausea.^88^ Clearly, more adverse side-­effects would be expected from infusion, but this may greatly enhance the host immune response against the tumour. Combined topical and infused drug delivery may enhance the efficacy of the current treatments. Conventional radiotherapy, chemotherapy, monoclonal antibodies, immunotherapy and OVs can be combined in multiple combinations to find the most efficacious therapy regimen.^89,90^

The application of nanoparticle viral delivery can protect the virus from the host immune system until it has infected the tumour; alternatively cell-based delivery of the OV using mesenchymal stem cells shows some promise for future treatments.^91^

### Viruses that have been explored as treatment options for melanoma

In two phase I trials, the prototypical development of the vaccinia virus JX-594 using poxvirus was modified through the deletion of virulence genes and insertion of the passenger cytokine gene human granulocyte-macrophage colony stimulating factor (GM-CSF).^56,60^ These modifications enable activation of local macrophages and dendritic cells; JX-594 was found to replicate successfully within the TME, resulting in local oncolysis with the additional benefit of remaining both safe and effective with increasing dosage.^56,60^ Another phase I trial using a vaccinia virus, encoding T-cell co-stimulating molecules, found that tumour regression could be associated with increased expression of cytokines and T cells; this intervention presented with low-grade toxicity and provided evidence of inducing systemic immunity.^92^ Tumour-specific cytotoxic T-cell activation is essential in mounting the immune response against the tumour and new ventures introducing bi- and trivalent antibodies specific for malignant cells can enhance this immune response.^93^

The herpes simplex virus (HSV-1) is an attractive contender for use in the treatment of melanoma as it has a large genome containing multiple nonessential genes than can be replaced with therapeutic transgenes, simultaneously reducing pathogenicity.^94^ T-VEC is the first success story of OV immunotherapy, being approved by the FDA for treating advanced or unresectable melanoma.^95^ Clinical trials phases I–III demonstrated positive outcomes using T-VEC in melanoma patients.^96–98^

HF10 is a spontaneously mutated strain of HSV-1 with two genetic alterations that have reduced the neuroinvasive nature of the virus, thus decreasing the risk of nervous system infection.^99^ HF10 is evidenced to infect and lyse murine and human malignant melanoma cells *in vitro*.^100^ This study also showed that intratumoral injection of HF10 into immunocompetent mice with malignant melanoma reduced not only the injected tumours, but distant metastases as well; this implies that the strain has capabilities of achieving direct oncolysis and initiation of a systemic antitumor immune response.^100^ When HF10 was used in combination with the chemotherapeutic drug dacarbazine, a vigorous systemic antitumour immune response was observed alongside prompt and potent cytotoxic effects relative to monotherapy, resulting in prolonged survival.^101^

Coxsackievirus A21 (CVA21) exhibited oncolytic effects in melanoma cells in preclinical studies.^102^ When studied in phase I and II clinical trials in patients with advanced, unresectable melanoma, intratumoral administration of CVA21 was well tolerated and with low-grade reactions.^103,104^ A concern regarding the therapeutic use of CVA21 stems from the possibility of patients having pre-existing immunity to the virus as it naturally occurs and circulates within communities.^105^ The use of coxsackieviruses A13, A15 and A18 is being explored as alternatives to CVA21 as protective antibodies were not detected within the patients with melanoma studied.^105^

The oncolytic action of the reovirus Reolysin^®^ was assessed in a phase II clinical trial whereby patients with metastatic melanoma received the OV intravenously.^106^ It was well tolerated, but with only 2 out of 13 patients evidencing viral replication, it does not serve as a strong candidate for use as a monotherapy in the treatment of metastatic melanoma.^106^ Despite not meeting its primary efficacy target, the trial data supports the use of reoviruses as a component of combinatorial strategies to treat malignant melanoma.^106^ An overview of some OV trials for melanoma patients is shown in Table 1.

**Table 1:** Shows some clinical trials of oncolytic viruses for melanoma patients

Clinical trial number	Clinical trial phase	Melanoma cancer stage	Intervention	Virus type	Outcome
NCT04152863	II	IV	Gebasaxturev (V937) + pembrolizumab intratumoral	Coxsackievirus A21	Improved ORR
NCT04303169	I/IIa	IV	Intratumoral	Coxsackievirus A21	Safe, improved ORR
NCT03190824	II	III–IV	Intratumoral, OBP-301 (telomelysin) with pembrolizumab	Type 5 adenovirus	Safe, improved ORR
NCT04291105	I/II	IIIB–IV	Intratumoral, VV1 + cemiplimab	Vesicular stomatitis virus (IFN-β)	Safe and effective
NCT03544723	II	Recurrent or metastatic-IV	AD-53 +ICI	Adenovirus p53	Reduced tumour size
NCT01397708	I/II	Unresectable, III–IV	AD engineered to express hIL-12	Adenovirus vector	Well tolerated improved survival rates
NCT04698187	II	Unresectable, metastatic-IV	CMP-001 (vidutolimod) + nivolumab	Virus-like particle, mimics bacterial DNA	Improved ORR
NCT04695977	II/III	Recurrent or metastatic	CMP-001 + nivolumab	Virus-like particle	Longer duration of response
NCT04401995	II	IIIB–D	CMP-001 + nivolumab	Virus-like particle	Pathological CR
NCT04708418	II	Resectable, III	CMP-001 + nivolumab versus Ab alone	Virus-like particle	Objective response
NCT03684785	Ib/II	Refractory, metastatic	Cavrotolimod, spherical nucleic acid	Nanoparticle Toll-like Receptor 9 Agonist	Reduced tumour burden
NCT02857569	I/II	Unresectable, IIIB–IVA	T-VEC, intratumoral ipilimumab + intravenous nivolumab	HSV, ipilimumab, nivolumab	Not published
NCT02423863	I/II	Unresectable, advanced	Poly-ICLC	dsRNA	Some clinical benefit
NCT02263508	III	IIIB–IV	T-VEC + pembrolizumab	Herpes Simplex (HSV)	PFS, OS
NCT02211131	II	Resectable IIIB–IVMIa	Neoadjuvant T-VEC	HSV	RFS
NCT02819843	II	IIIB–IV	T-VEC	HSV	Response
NCT02288897	III	IIIB–IVMIa	T-VEC	HSV	PFS
NCT02509507	I	IVMIc	T-VEC	HSV	Safety, improved outcomes, high CD8^+^
NCT02014441	II	IIIB–IV	T-VEC	HSV	T-VEC DNA in blood or urine
NCT02366195	II	IIIB–IV	T-VEC	HSV	Intratumoral CD8^+^ and ORR

The tumour staging is included, as well as the type of virus stated, some patient outcomes and clinical trial phases. AD, adenoviral p53 gene therapy; CR, complete response; dsRNA, double-stranded RNA; HSV, herpes simplex virus; ICI, immune checkpoint inhibitor; IFN-β, interferon β; ORR, objective response rate; OS, overall survival; PFS, progression-free survival; poly-ICLC, a synthetic complex of RNA, poly L-Lycine and carboxymethylcellulose; RFS, recurrence-free survival; T-VEC, Talimogene Laherparepvec.^107,108^

### T-VEC as monotherapy in the treatment of melanoma

The first human study of T-VEC was a phase I trial observing the viral replication, GM-CSF expression and tumour necrosis in patients with refractory cutaneous or subcutaneous metastases from melanoma.^96^ Predominantly low-grade toxicity was reported, and four patients exhibited signs of systemic immune response.^96^ Next was a phase II trial examining the effect of T-VEC in patients with unresectable or metastatic melanoma, which had an overall objective response rate (ORR) of 26% with a 1-year survival rate of 58% and mild side-effects experienced by 85% of patients.^97^ In the subsequent phase III (OPTiM) trial, 436 patients with locally advanced or metastatic melanoma were enrolled.^98^ The primary outcome measure was the durable response rate (DRR) which describes the percentage of patients experiencing a complete or partial response lasting ≥6 months and beginning within the first 12 months of receiving treatment.^98^ When compared with the performance of GM-CSF alone, the DRR for T-VEC monotherapy was 14% higher, however the ORR remained as 26% in phase III and survival data are not available.^98^ These trials highlight the efficacy of T-VEC delivery as a monotherapy in terms of its direct oncolytic effect and ability to generate responses in distant, non-injected tumours, suggesting activation of an immune response against tumour-associated antigens.^96–98^ However, limitations arise with respect to the lack of responses observed in stage IV1b–1c melanoma patients presenting with lung or visceral metastases compared with patients with stage IIIB–IV1a melanoma—the ORR for stage IIIB–IV1a was 41% compared with 9% in stage IV1b–1c; this may restrict the suitability of T-VEC as a monotherapy to stage IIIB–IV1a.^109^ On the other hand, retrospective analysis of data from the phase III trial recognized a durable complete response in rare and aggressive inflammatory melanoma.^110^ The design of phase III trial does warrant a further investigation into the efficacy of mono-therapeutic T-VEC, as the comparison arm is not representative of the current state of the art treatment for advanced melanoma, nor did it factor in a combination approach during that period of time.^98^

### T-VEC used as a part of combination therapy in the treatment of melanoma

Using mouse melanoma models in preclinical studies, the combination of intratumoral T-VEC and anti-CTLA4 monoclonal antibodies (ipilimumab) was evaluated in order to gain a better understanding of the local and systemic anti-tumour immune responses initiated.^111^ The combination therapy resulted in all injected tumours and half of non-injected tumours being cured in the mice.^51^ This revealed a significant increase in T cells within injected and non-injected tumours; these T cells were found to be tumour-specific.^111^ Following this, a randomized phase Ib/II study compared the combination of T-VEC with ipilimumab, against ipilimumab alone in unresectable stage IIIB–IIIC and IV melanoma patients, generating a significantly higher ORR for the combination arm relative to ipilimumab alone for all melanoma stages studied.^112^ In this study, BRAF mutational status was also considered.^112^  *BRAF* wild-type tumours were found to have a greater magnitude of efficacy with the combination treatment, with an ORR of 42% in contrast to the ORR of 10% for the ipilimumab arm.^112^ To compare, *BRAF* mutation–positive tumours responded with an ORR of 34% when receiving the combination treatment and 32% when receiving ipilimumab alone.^112^ This reveals a gap in the research in terms of considering whether the mutational status of a tumour has bearing on the reaction to the OV.

In a phase Ib/III trial, the combination of T-VEC with the anti-PD-1 antibody pembrolizumab is also being tested in patients who have not previously received treatment and present with stage IIIB to IV melanoma.^113^ The preceding phase Ib trial for this combination produced an ORR of 61.9% and a complete response (CR) rate of 33.3%.^113^ An increase in the ORR to 67% was noted after the data cut-off, with the status of two patients changing from partial response to complete remission, raising the CR rate to 43%.^114^

A recent clinical phase III trial of T-VEC and pembrolizumab unfortunately did not show a significant improvement, when compared with placebo-pembrolizumab therapy, despite the good treatment response rates (ORR, DRR) and the lack of additional toxicities. The encouraging phase I trial results were not reflected in this recent phase III trial, unfortunately with significant improvement in progression-free survival and overall survival. This combination continues to be investigated and may show improved results in the future interventions.^115,116^

### The potential of oncolytic viruses in treating uveal melanoma

A study using a murine model showed that the oncolytic HSV-1-EGFP (enhanced green fluorescent protein) displayed signs of initiating both a local and systemic antitumor innate immune response.^117^ Both uveal and subcutaneous tumours were found to be reduced in size, implying the direct action of oncolysis and demonstrating the OV’s capability of initiating an immune response to target distant tumours.^117^ Another study using 3D melanoma cultures found conflicting results, in that HSV-1 was initially found to significantly decrease the quantity of tumour cells but did not result in complete elimination.^118^ Over a 17-day observation period, HSV-1 was found to promote the growth of some melanoma cells after inoculation with the virus.^118^

## Challenges encountered in OV treatments

Despite the success of animal models in the field of OV therapy for cancer, the impact in the clinical environment has been disappointing for multifactorial reasons. There are multiple and complex tumour interactions surrounding the tumour (TME), the host immune response and the virus infection. The ability of the virus to cause oncolysis is affected by the mode of delivery, viral tropism and the distribution of the virus.^119^ Acquiring the effective dosing level in the therapy is a key factor in OV therapy, which may vary between patients. Also, the degree of viral penetration into the tumour achieved by the OV infection ensures effective eradication of the tumour. In the case of melanoma direct injection into the superficial tumour enhances the penetration but some tumours are less accessible for this degree of penetration. Viral infiltration can also be impaired by the TME where abnormal vascular hyperpermeability can cause interstitial hypertension and aberrant lymphatic networks within the solid tumour mass.^119,120^

The host immune system may have established some antiviral immunity to the OV due to an adaptive response from a previous infection to the virus species or crossreactivity with another virus. Also, the heterogeneity of tumours involving genetic mutations and cell types can affect the ability of the virus to infect the tumour cell.^47,119^

**Figure 2:**
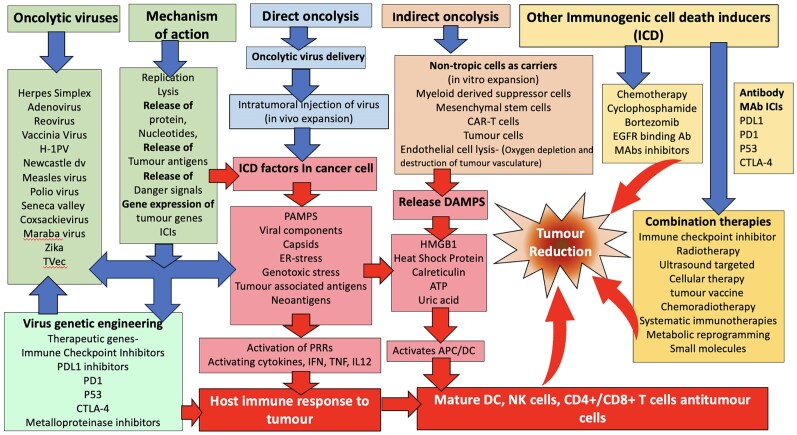
Methods of oncolytic virus (OV) delivery and mechanisms compared or combined with other established therapies. The table includes examples of possible viruses, genetic engineering to adapt their efficacy and mechanism of action. Methods of delivery such as direct delivery of intratumoral injection are described, and indirect methods of delivery are explored. Some therapies target endothelial cells to damage vascular circulation and oxygenation of tumour cells. Ab, antibody; APC, antigen-presenting cell; ATP, adenosine triphosphate; CAR-T, chimeric antigen receptor T cell; CTLA-4, cytotoxic T lymphocyte-associated protein 4; DAMP, damage-associated molecular pattern; DC, dendritic cell; EGFR, epidermal growth factor receptor; ER, endoplasmic reticulum; HMGB1, high mobility group box 1 protein; ICD, immunogenic cell death inducer; ICI, immune checkpoint inhibitor; IFN, interferon; IL12, interleukin-12; mAb, monoclonal antibody; NK, natural killer; PAMP, pathogen-associated molecular patten; PD1, programmed cell death protein 1; PDL1, programmed death-ligand 1; PRR, pattern recognition receptor; TNF, tumour necrosis factor.

The selection of suitable patients for this therapy is important, where immunocompromised individuals may not be suitable candidates for OV therapies due to the OV genetic manipulation that may result in some unexpected toxic outcomes of alternative viral targets or may result in a cytotoxic gene expression.^119^ Some viruses could mutate or recombine *in vivo* or evolve into transmissible viruses. Thus improved safety and security of OV design and usage requires further development. The host immune response to both virus and tumour needs to be further enhanced and the ability of the virus to replicate effectively in the tumour needs to be optimal and improved to enhance the efficacy of OV cancer therapy. The optimum engineering and combination of OVs is likely to improve treatment strategies and the quality of life of cancer patients in the future.^120^ The insertion of ICIs, antibody, antiangiogenic and metalloproteinase inhibitors can enhance the survival of the OV and tumour cell death (Figure 2).^119,120^

There are some other key factors to consider when exploring novel strategies that improve optimum delivery of OVs, with highly tropic targeting of tumour tissue while avoiding unexpected and unwelcomed interactions with normal tissue and physiology. Some examples of engineered oncolytic viruses are ONYX-15 and H101, which prevent viral replication in normal cells by expressing E1B. The virus HSV JS1 was found to enhance the host immune response to the virus, and Reolysin (Pelareorep) is an RNA virus that targets cancer cells that have an activated renin–angiotensin system (RAS) pathway.^121^

In order for an OV to evade the host immune response it must evade antiviral cytokines, complement activation and macrophages all of which could rapidly clear the virus in the host. Other barriers to the success of an OV includes tumour necrosis, interstitial hydrostatic pressure, fibrosis and tumour extracellular matrix, in addition to interferon antiviral tumour responses.^121^

## Future perspectives for melanoma therapy using OVs

It cannot be denied that some melanomas are immunotherapy refractive, and new strategies of drug combinations and delivery are required for these patients. T-VEC (Imlytic) remains the main registered oncolytic therapy for cancer, but many different engineered versions of oncolytic viruses are being developed and trialled as vehicles for immunogenic elements and antitumour activity. The tumour immune landscape is crucial to the existence and growth of a tumour and transforming the TME to be inflammatory against the cancer cells is a growing area of interest in adapting current oncolytic strategies. Combinations of ICI, particularly monoclonal antibodies to ICI receptors, like anti-PD-L1 and CTLA4, are currently enhancing some OV cancer treatment potential in clinical trials.^122,123^ Another consideration to include as combination factors with OVs is angiogenesis inhibitors, both as transfected elements in the virus design and separate delivery of drugs known to prevent new blood vessels to the tumour.

HSV, in T-VEC, is tropic to oral epithelium, however herpes zoster virus is specifically tropic to skin so genetically engineering this virus, while inhibiting its pathogenic characteristics, may hijack its skin-tropic nature and be an interesting approach to treating skin cancer. The availability of CRISPR/Cas9 and bacterial artificial chromosomes^124,125^ to redesign a virus genotype means that the possibilities for successful oncolytic viral immunotherapies are endless and much work is to be done to harness these effectively, translating from bench to bedside.

A genetically modified version of the polio virus has been trialled as an OV to treat melanoma as the polio receptor (CD155) is upregulated on melanoma cells.^126^ PVSRIPO is an oncolytic polio: rhinovirus recombinant and can be introduced intratumorally, and induce innate inflammatory anti-tumour activity, particularly.^127^ The initial success of animal studies has shown that PVSRIPO propagates well and is cytotoxic to cancer cells. This initial success in lysing tumour cells may be because melanocytes originate from the embryonic neural crest.^127^ Although melanomas are often found on skin and mucosa, neural crest cells can migrate to the gastrointestinal tract and brain.

Reovirus is a benign RNA virus that specifically targets transformed cells with activated RAS pathways, and RAS genes are often mutated in cancer cells, which can be targeted by recombinant reoviruses.^89^ In combination with conventional cancer therapies, OVs of reoviruses are a promising option to target mutated cancer cells.

Many innovative investigations are underway that experiment with genetically transformed OVs that only usually infect animals in their native form, not humans, so the host immune system does not attempt to neutralize these pathogens when they target the tumour, while mounting an anti-tumour inflammatory response.^128^ Examples of these are recombinant versions of Newcastle virus, which infects pigeons, and swine vesicular disease.^129–131^

Are there novel specific mutational targets in the tumour and can tropism to these be engineered into OVs? Due to the high rate of both intra-heterogeneous and inter-heterogeneous cancer cell transformation, a personalized approach of individual patient-specific therapies is expected to enhance treatment outcomes for malignant melanoma patients.^132^

Many inhibitory factors can be introduced into the genetic design of an OV, including factors that inhibit cell proliferation like the ICI enhancing apoptosis in cancer cells. Genetic insertions that enhance inflammatory factors that alert the host immune system such as DAMPs and PAMPs associated with mutated cellular DNA. Another current proposal is the inclusion of anti-angiogenic factors either as genomic insertions into the OV or as a trans method in combination with angiogenesis blocking monoclonal antibodies.

This review captures the potential of using oncolytic viruses in the treatment of cutaneous melanoma through its investigation of the different types of viruses that have been studied and through exploring T-VEC as a monotherapy or used in combination with other treatments. Understanding the mechanisms by which OVs operate is essential in establishing their efficacy in not only targeting the tumour cells directly, but through indirect mechanisms and by instigating an immune response to the cancer. There are several viruses, treatment combinations and methods of delivery that have shown promise as prospective OV treatments, however future studies are required to corroborate this.

When compared with cutaneous melanoma, the current research into the application of OV treatment to uveal melanoma, is relatively thin, creating a gap in the research that should be addressed in future studies. Overall, the literature provides substantial evidence that OVs are effective in treating cutaneous melanoma, but there are opportunities for improvements such as finding a more effective delivery method for inaccessible tumours, considering different treatment combinations using OVs and understanding the influence that tumour gene mutational status has on the cancer’s reaction to treatment.

To conclude, it is clear that due to their high killing capability, OVs are efficient at destroying tumours, can be targeted accurately to tumours with few adverse reactions and can be combined with several different mechanisms for tumour destruction.

## Data Availability

Data sharing is not applicable to this article as no new data were created or analysed in this study.
